# Memristor-based PUF for lightweight cryptographic randomness

**DOI:** 10.1038/s41598-022-11240-6

**Published:** 2022-05-23

**Authors:** Hebatallah M. Ibrahim, Heba Abunahla, Baker Mohammad, Hoda AlKhzaimi

**Affiliations:** 1 Center for Cyber Security, New York University Abu Dhabi, Abu Dhabi, UAE; 2 System on Chip Center and Electrical Engineering and Computer Science, Khalifa University, Abu Dhabi, UAE

**Keywords:** Electrical and electronic engineering, Computer science

## Abstract

Physical unclonable functions (PUF) are cryptographic primitives employed to generate true and intrinsic randomness which is critical for cryptographic and secure applications. Thus, the PUF output (response) has properties that can be utilized in building a true random number generator (TRNG) for security applications. The most popular PUF architectures are transistor-based and they focus on exploiting the uncontrollable process variations in conventional CMOS fabrication technology. Recent development in emerging technology such as memristor-based models provides an opportunity to achieve a robust and lightweight PUF architecture. Memristor-based PUF has proven to be more resilient to attacks such as hardware reverse engineering attacks. In this paper, we design a lightweight and low-cost memristor PUF and verify it against cryptographic randomness tests achieving a unique, reliable, irreversible random sequence output. The current research demonstrates the architecture of a low-cost, high endurance Cu/HfO$_2/p^{++}$Si memristor-based PUF (MR-PUF) which is compatible with advanced CMOS technologies. This paper explores the 15 NIST cryptographic randomness tests that have been applied to our Cu/HfO$_2/p^{++}$Si MR-PUF. Moreover, security properties such as uniformity, uniqueness, and repeatability of our MR-PUF have been tested in this paper and validated. Additionally, this paper explores the applicability of our MR-PUF on block ciphers to improve the randomness achieved within the encryption process. Our MR-PUF has been used on block ciphers to construct a TRNG cipher block that successfully passed the NIST tests. Additionally, this paper investigated MR-PUF within a new authenticated key exchange and mutual authentication protocol between the head-end system (HES) and smart meters (SM)s in an advanced metering infrastructure (AMI) for smartgrids. The authenticated key exchange protocol utilized within the AMI was verified in this paper to meet the essential security when it comes to randomness by successfully passing the NIST tests without a post-processing algorithm.

## Introduction

Cryptographic Randomness is an essential property to maintain when it comes to building confidentiality, authentication and integrity-focused primitives, and security solutions. For example for encryption primitives, random numbers are used in both symmetric and asymmetric encryption algorithms to generate initial values, nonces, cryptographic keys, and round constants among other purposes^1^.

Thus, random number generators (RNG) are an important resource in many areas, yet producing random numbers is challenging as selecting a specific source of randomness governs the quality, security, and robustness of the resulting output. For example, it is important to understand whether the random number generator is non-deterministic (True) RNG or the deterministic (Pseudo) RNG^2^. The difference is significant, since, by definition, the output of a true random generator cannot be tampered with, whereas Pseudo random generators produce a sequence of numbers that can be reproduced at a later date if the starting point in the sequence is known^3^. Today, true random numbers are most critically required in cryptography and its numerous applications to cyber-security, especially interactive lightweight focused systems, such as Smart Energy Grid, e-banking, internet trade, prepaid cards, etc.

Cryptographic material, such as Digital keys, are conventionally saved in memories for cryptographic applications. However, digital memories are at risk of physical attacks. Complex and costly tamper-proofing mechanisms have to be implemented in hardware to secure these cryptographic materials. In 2001 Pappu^4^ proposed physical one-way functions, known as PUFs nowadays, to act as refined primitives to generate true intrinsic randomness which is critical for cryptographic applications. The properties generated can be used to enhance the security characteristics of applications by providing better confidentiality and authentication attributes through enhancing randomness. More importantly, they are less complex to fabricate, inexpensive, particularly difficult to duplicate, admit no compact mathematical representation to be reversed, and they do not need to be saved on memory, thus, they are more resistant to physical attacks. The Physical Unclonable function we propose in this paper is a lightweight, efficient, unique, robust, and uniform low cost security primitive, which exploits the intrinsic variation of the delay time as the source of randomness to build a TRNG unit that employs our HfO$_{2-x}$ memristor only. Memristor devices are considered low power devices due to their zero-leakage current and low switching time. Thus, memristor-based PUFs are considered low energy systems compared to conventional CMOS-based PUFs^5^. Previous work proposed on Memristor-based PUFs has proposed several models verifying the efficiency through demonstrating different cryptographic randomness tests such as uniformity, uniqueness, diffuseness, and repeatability. However, few were able to be pass the national institute of standards and technology (NIST) tests. Others couldn’t avoid a complex design and didn’t consider the cost factor. Also, few verified the functionality and reliability of their proposed PUF in actual systems or protocols.

In our paper, the main contributions can be summarized as follows: (a) We propose a simple design, low-cost, reliable memristor based PUF (MR-PUF) that successfully achieved true random binary sequence. (b) Additionally, our MR-PUF responses were tested using NIST SP 800-22 statistical tests and other cryptographic randomness tests to verify its true randomness properties. (c) Furthermore, we introduced two different cryptographic and security level applications of the proposed MR-PUF. We were able to verify the reliability and randomness of our MR-PUF through integrating it in two applications block-cipher design and advanced metering infrastructure (AMI). (d) Finally, we have introduced general testing mechanisms and simulations to the application proposed.

In this paper, we start by presenting the PUFs background in the coming section, as well as the limitation of memristor-based PUFs presented in the literature review and some compilation of cryptographic randomness tests in. Then explain the memristor performance and functionality through discussing the switching behavior in and demonstrate our proposed memristor model fabrication process and its switching behavior. After that we verify the randomness of our MR-PUF in section using NIST tests and verifying the output uniformity, diffuseness, uniqueness tests, and repeatiability. Finally, we verify the feasibility and reliability of our MR-PUF in two systems, block-ciphers chain model to generate randomness and AMI model to enhance the security of the mutual authentication protocol. Then, conclude with the conclusion and future work.

## Background

In applications where security is essential such as communication protocols TLS/SSL/HTTPS, contactless smartcards, e-banking, internet trade, etc, they require lightweight, secure and efficient cryptographic primitives to create a secure communication regardless of any malicious presence. For example, the key generation module is considered one of the most critical parts of the encryption crypto-system where keys are created using random number generators (RNGs). The two commonly known categories for the RNGs are deterministic (Pseduo-Random) RNGs and unpredictable (True) RNGs as shown in Table 1. Several Pseudo random techniques are supported by theories and have produced very good results. However, Pseudo RNGs are algorithms that use mathematical formulas or simply precalculated tables to produce sequences of numbers that appear random. However, they can be tampered with using the preceding outputs or the initial state (seed), by definition such generators are not random. Realistically, PRNG’s feature a perfect balance between 0’s and 1’s (zero bias) but also strong long-range correlations which undermine cryptographic strength and can show up as unexpected errors in Monte Carlo calculations and modeling^6^.

To ensure the security of the cryptographic primitives we need to ensure that the randomness source is truly random otherwise the whole system will collapse. True random number generators (TRNGs) are required to ensure the security of crypto-systems. TRNGs extract randomness from physical phenomena, the physical phenomenon used is a quantum phenomenon or a phenomenon with chaotic behavior (such as memristors and silicon cavities). The challenge is to retain the TRNG cryptographic characteristics, there have been many proposals in the literature that can be considered realistic in specific terms based on the time and memory complexity of the attacks that can be implemented to compromise the proposed characteristics. Hardware solutions as explained in the paper, are considered one of the most yet unrefined proposed solutions to get closer to implementing practical TRNGs. There are several examples of the hardware PUFs in the literature that have been built in lab. However, the unique approach of our MR-PUF is that we have fabricated the chemical characteristics of the PUF in the lab in order to obtain ideal cryptographic results as illustrated in the tests we have produced in the paper.

There are several metrics to evaluate PUF performance. Randomness, uniqueness, and uniformity are the three most-used metrics among them^7^. PUFs exploit the intrinsic quantum complexity and uniqueness of physical systems to generate secure random signatures.

**Table 1:** TRNG versus PRNG.

RNG	TRNG	PRNG
Determinism	Unpredictable	Deterministic
Periodicity	Not Periodic	Periodic

### Physical unclonable functions (PUFs)

PUFs extract unique sequences from unpredictable and uncontrollable process variations during IC manufacturing. The digital keys are confidential within its structure. Any invasive or semi-invasive attack will destroy the chip’s physical structure. A major advantage of PUFs is that they are easy and inexpensive to be built but impossible to duplicate because they rely on uncontrollable physical parameter variations that occur during the hardware device manufacture^8^. Most importantly, the PUF signature is only derived from the intrinsic complexity of the physical device when it is needed and vanishes otherwise, every time a given challenge (input) is presented to a PUF, a corresponding response (output) is given. Therefore, there is no need for digital memories, which makes PUFs invulnerable to hardware attacks. This response generated by a PUF is based on a complex physical function that is unique to each PUF. If a given challenge is given to several PUFs with the same design, different responses will be produced. The challenge and its corresponding response are called (CRP). A set of CRPs can be treated as a fingerprint of the PUF^9^.

Traditional PUFs are CMOS-based such as Arbiter PUF (APUF)^9^, Ring Oscillator PUF (ROPUF)^10^, SRAM (Static Random Access Memory) PUF^11^. They exploit uncontrollable process variations in conventional CMOS fabrication technology. CMOS-based PUF can produce chip-unique signature based on the intrinsic variations, that varies randomly from one chip to another^12^. Due to fabrication variations, there are random delay differences on symmetrical electrical paths on a chip. The randomness of the delays is sufficient to ensure a unique PUF response for each individual device instance^13^. These variations are translated into bits of information unique to each device. These bits can be employed in different categories of security protocols, such as secret keys, public keys authentications^14^, RFID tags^15^, IP protections^16^, IC piracy^17^, unique identifiers and pseudo random generators^18^.

Generally, there are two main applications of PUFs which are authentication and secure key generation. Based on the two applications, the PUFs are generally categorized as “strong PUFs” and “weak PUFs”. Strong PUFs can be targeted for authentication, while weak PUFs are more fit for the key generation.

**Table 2:** Strong versus weak PUF.

PUF types	Strong PUF	Weak PUF
CRPs	Large number	Small number
Main Applications	IC identification,Key generation	Key generation
Common PUFs	Arbiter, RO	SRAM, Latch, Butterfly

- Strong PUFs are chaotic physical units with a complex challenge-response behavior characterized by large (CRPs)^19^. It is impossible to physically clone a strong PUF and impossible to measure or determine all the CRPs for a strong PUF within a limited time. Typical examples for the strong PUFs are: the arbiter PUF^9^ and the ring oscillator (RO) PUF^10^ as shown in Table 2. In contrast to the strong PUFs, the weak PUFs may have very few CRPs.
- Weak PUFs can be considered as a distinctive form of memory, however, they are more resilient to invasive attacks than the non-volatile memory like EEPROM^20^. The most typical weak PUFs are the memory-based PUFs: SRAM PUF^21^, latch PUF^22^, and butterfly PUF^23^.

Current PUF designs face several challenges, such as extensive CRP access attacks to PUFs that acquire a limited number of CRPs, model building attacks^19^, reliability deterioration due to environmental conditions that are rarely due to aging^24^. Therefore, the design of superior PUFs that maintains a suitable trade-off between quality and area overhead, remains a research aim. Most recent PUF technologies are discussed in literature to mitigate some of the overhead and performance-related shortcomings.

Here in this paper, we exploit the unique properties of Nano-electronics rather than CMOS technology to provide an opportunity for building a PUF design that addresses the limited number of CRPs, model building attacks, reliability deterioration, and less utilization area. More importantly, achieving uniqueness, uniformity, irreversibility, and low cost which are critical for security^25^. Memristor PUFs have proven to be more resilient to attacks such as reverse engineering^26^. Several studies have been proposing memristor PUFs due to the inherent randomness at both the memristor level, due to the C2C programming variation of the device, and the fabrication process level such as the cross-sectional area and variations. It is clear that the generated characteristics are not identical which allows extracting unique keys, thus, the user will not be able to control its resistance. Leveraging this phenomenon, our MR-PUF can achieve a unique, reliable, irreversible PUF signature^27^.

### Limitations of previously proposed memristor design for hardware security

In this section, we are revisiting similar designs available in the literature and drawing on the added value that our research is highlighting.

The design for a memristor-based (TRNG) has been discussed in literature and some designs have been tested the several NIST statistical randomness tests. However, not all have proven to pass all the 15 NIST tests. In^28^ the author proposed a memristive read and write PUF. Two Al/CUxO/Cu devices were implemented, they demonstrated lateral switching wherein, one of the two devices became fixed in an LRS state. No further tests were applied to the other working device. In^29^ the author continued the work on^28^ and presented N-bit read and write Memristive PUF (M-PUF) and verified its efficiency through demonstrating the uniqueness, uniformity, and bit-aliasing to measure the statistical quality of the M-PUF. Hybrid memristor-CMOS PUF circuits is proposed in^30^, benefiting in less design overhead than CMOS-only PUFs. They exploited the delay variation in the memristor devices to generate instance-specific signatures. They tested the reliability, uniqueness, and uniformity of the different sized PUFs. The authors also tested the reliability of the PUFs under different temperatures and voltages. However, they did not ensure its randomness and applied the 15 NIST tests. In^26^, the authors introduced a re-configurable PUF (rPUF) without additional hardware. rPUFs are needed for application required revocation or updated secure key. The author demonstrated the efficiency and security of their PUFs by demonstrating the uniqueness, reliability and a large number of challenge-response pairs (CRPs) through exploiting large information density available in nanocrossbar architectures. Memristors, or resistive switching devices, have been presented for a broad spectrum of applications because of their unique properties, such as low power consumption, fast switching speed, high endurance, excellent scalability, and CMOS compatibility. For non-volatile memories, the intrinsic variation in memristor switching parameters is a major challenge. However, this random behavior can be exploited in stochastic computing and hardware security applications. The author in^31^ exploited the randomness of the telegraph noise (RTN) from the low resistance state of a W/TiN/TiON/SiO$_2$/Si memristor. The resulting circuit demonstrated that the probabilities of “0” and “1” were highly dependent on the applied voltages, thus, the circuit is challenging to activate and control.

In^32^, the authors proposed a TRNG based on Cu/AlO$_x$ and Ti/HfO$_x$ memristors, using cycle to cycle (C2C) and device to device voltage variations. The proposed memristive devices were non-volatile, requiring SET-RESET pulses for each output bit, and careful tuning of the applied voltage. None of the previous memristor-based TRNGs passed all the 15 NIST 800-22 statistical randomness tests even with post-processing of data, leaving the claimed true nature of the randomness debatable. In 2016, the authors of^33^ demonstrated a TRNG that passed the 15 NIST tests using randomness from a small current fluctuation at certain resistance states in TaO$_x$-based devices. However, complicated algorithms and costly circuits were needed to ensure the quality of generated binary bits. In 2017, Pt/Ag/Ag:SiO$_2$/Pt memristor device was presented in^34^, the authors verified their TRNG Diffusive memristor (D-Memristor) by passing the 15 NIST test without the need for post-processing algorithm. However, the authors used five stacked layers consisting of Pt/Au/Ag/SiO$_2$/Pt presenting a complex structure compared to the three layers used in our work. This leads to a higher cost due to the increased material amount and the number of fabrication steps of the device. Also, the device in^34^ includes Au, Ag and Pt, which have higher cost compared to the copper electrodes used in our device. Thus, the device proposed in^34^ has a complex structure and higher cost. The security or practicality limitations of the previous proposed PUFs in terms of the number of NIST 800-22 tests that have been passed is summarized in Table 3. The memristor we propose in this paper is an efficient, unique, robust and uniform low cost security primitive. The Cu/HfO$_{2-x}/p^{++}$Si devices are fabricated using a low-cost sol–gel spin-coating method. It is based on HfO$_{2-x}$ which is compatible with advanced CMOS technologies and have high endurance. Moreover, the fine fabrication (in nm) of the HfO$_{2-x}$ layer as a switching medium provide fast switching speed. The authors in^35^ proposed Resistive RAM (RRAM) TRNG based on HfO$_{2-x}$. However, their security design only passed 12 tests out of the standard 15 NIST tests. In our design, we used the intrinsic variations of the delay time as the source of randomness to build a TRNG device. Binary bit sequences generated by our proposed MR-PUF passed all the 15 NIST Special Publication 800-22 randomness tests without any additional algorithms ensuring the enhanced security our design provides. Additionally, Cu/HfO$_{2-x}/p^{++}$Si MR-PUF is a cost-efficient approach to further improve the bit generation randomness and have never been exploited in literature, to the best of our knowledge.

**Table 3:** Comparison between the proposed Memristor design and the relevant Memristors presented in literature.

Relevant TRNGs/PUFs designs in the literature	Performed NIST tests	Authors/references
Memristive read and write PUF	–N/A–	^28^
N-bit read and write Memristive PUF (M-PUF)	–N/A–	^29^
Hybrid memristor-CMOS PUF	–N/A–	^30^
Nanocrossbar memristor PUF	–N/A–	^26^
W/TiN/TiON/SiO2/Si memristor	–N/A–	^31^
Cu/AlOx and Ti/HfOx memristors	–N/A–	^32^
TaOx-based devices	All 15 NIST tests	^33^
	(expensive quality bits generated)	
Pt/Ag/Ag:SiO2/Pt memristor	All 15 NIST tests (complex device Structure)	^34^
	(complex device Structure)	
RRAM TRNGs	12 NIST tests	^35^
Cu/HfO$_{2-x}/p^{++}$Si Memristor	All 15 combined with the literature	MR-PUF TRNG proposed in this paper
	three additional tests using efficient and low cost structure	

### Cryptographic randomness testing

The quality of the random numbers for a cryptographic system evaluates the security strength of the system. The randomness is measured by using tests suited for evaluating true random bit generators intended for cryptographic applications. Most randomness tests evaluate one or more statistical properties of long sequences of random numbers, for example, bias, serial auto-correlation etc^6^. Some compilation of tests are more adjusted towards problems in PRNG’s (eg. DIEHARD^36^) some more to hardware RNG’s (eg. ENT^37^). The unfortunate fact is that these tests contain errors discovered later^38,39^. NIST 800-22^40^ is a package of 15 tests that were formulated to evaluate the randomness of bit sequences produced by either hardware or software cryptographic systems. NIST 800-22^40^ doesn’t offer guidance on how to implement TRNG or how to pass the tests, nonetheless, passing the 15 NIST 800-22 tests is vital for commercial use. In this section, we will investigate the NIST 800-22 testing map that we will use to test our TRNG. NIST^40^ is a set of statistical tests that are formulated to test a specific null hypothesis (H0). The H0 under test is that the sequence being tested is random. Corresponding with the null hypothesis is the alternative hypothesis (Ha), which points that the sequence is not random. For each applied test, a conclusion is derived that supports or rejects the null hypothesis, thus evaluating whether the generator is producing random values or not.

The NIST Statistical Test Suite (Special Publication 800-22)^40^ contains 15 tests that evaluates the randomness of a binary sequence and each test targets a specific aspect of parameters. As per NIST 800-22 the fail and pass threshold are formally defined within the P values that we have used across the security analysis to define the success and the failure of the tests as depicted in the paper. The $\alpha$ which is the level of significance is one of the most important parameters in the test. If $\alpha$ is 0 that means that the randomness of numbers to be tested has a confidence value of 99%. Another vital parameter is the *p* value and it is the measure of randomness. If this value is equal to 1, numbers are said to have perfect randomness. If *p* value is less than 0.01, numbers are not random. Thus, the bits sequence is considered to be random if and only if the *P* value $\ge$ 0.01 and the pass rate exceeds the minimum pass rate for each test. In summary, for a fixed significance level or threshold a specific representation of *P* values will indicate a failure. For example, if the significance level is chosen to be 0.01 (i.e., = 0.01), then about 1% of the sequences are expected to fail. This indicates that a sequence passes a statistical test if the *P* value $\ge$ 0.01 and fails otherwise.

## Memristor model

In this section, we are introducing the details of the hardware design of the memristor that we have fabricated and discussing its unique hardware properties. A clear advantage of Memristor PUFs is the reduction in area utilization and the low energy consumption compared to CMOS-based PUFs. Our memristor based on HfO$_{2-x}$ as a switching medium is a favorable candidate given that HfO$_{2-x}$ provides high endurance due to the material’s high stability^41^. We have utilized sets of cycles to generate a random sequence and perform the one million bit tests. This device has high endurance and acquire fast switching speed due to the fine thickness (in nm) of the deposited HfO$_2$ layer. Additionally, our HfO$_{2-x}$ based memristor is compatible with advanced CMOS technologies, has a low cost and a simple synthesis process due to the only three stacked layers used in the fabrication process.

Furthermore, the memristor has a unique phenomenon that can achieve the uniqueness, irreversibility, and reliability required for efficient PUF designs called cycle-to-cycle (C2C) variation, meaning that every time the memristor cell gives different resistance than the previous time; depending on the previous current that passed through the cell. Thus, memristors have inherent randomness at both the memristor device level due to the C2C characteristic and the intrinsic variations of the device fabrication process level (such as thickness and cross-sectional area variations). Memristor-based TRNG can be used in numerous algorithms and protocols which use random numbers for the construction of encryption and decryption keys, initialization vectors, one time passwords, padding, nonces, and many more applications. In this paper, we integrated MR-PUF in AMI infrastructure and ciphers design to ensure the reliability of our proposed MR-PUF in different applications. The design for a memristor-based true random number generator (TRNG) has been discussed in literature^26–28,30^ and some designs have been tested by several statistical randomness tests designed by NIST. However, not all have proven to pass the NIST 15 tests.

### Switching behavior

**Figure 1:**
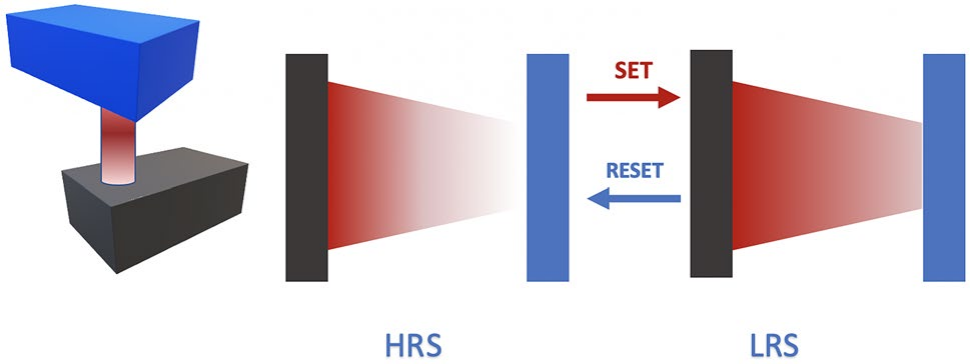
Memristor operation.

Figure 1 illustrates the operation principles of our MR-PUF. The concentration gradient of ions that can be moved back and forth using an applied electric field are the SET and RESET switching phases^26^ . The memristive device switches from HRS (High Resistance State) to LRS (Low Resistance State) with a positive potential difference between the bottom electrode and top electrode corresponding to SET switching. It switches from LRS to HRS with a negative potential difference between the bottom electrode and top electrode corresponding to RESET switching. When a memristive unit is programmed its memristance does not change even if its power supply is out except if a voltage higher than the threshold voltage is applied across the device^26^.

**Figure 2:**
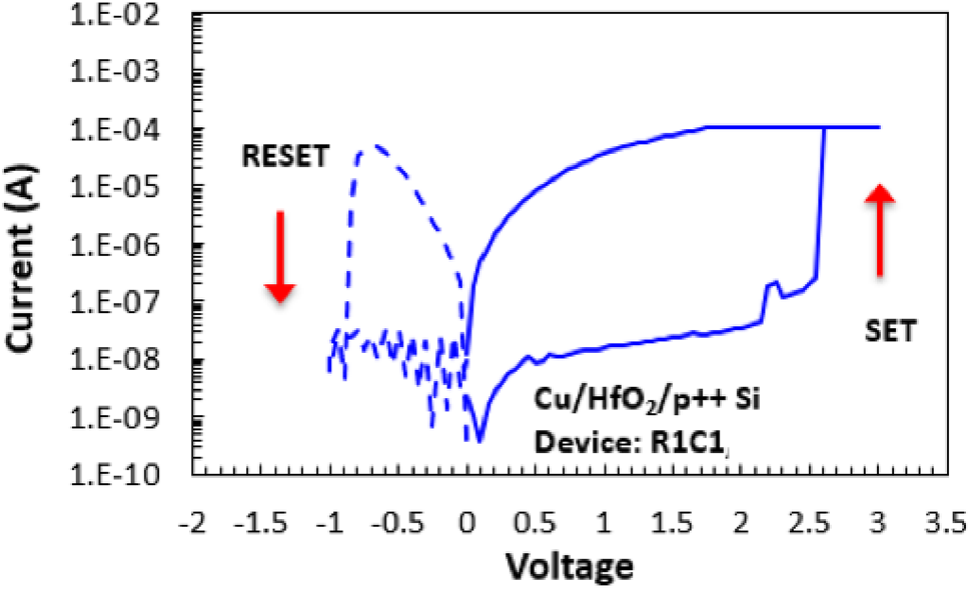
I–V characteristic of memristor during 1 Cycle (SET/RESET).

In Fig. 2, the electrical behavior of the fabricated MR-PUF is investigated to understand the switching mechanism of the device. The device starts with a high resistance state, and under the application of +3 V voltage bias, as shown in Fig. 2, a sharp jump in the current occurs at 2.5 V until it reaches the compliance current of 100 $\upmu$A. This operation is called SET, where one or more filaments are created to allow the current to pass through the device. A low compliance current is essential to reduce the power consumed by the device and to avoid reaching extremely high current levels which may permanently put the device in the low resistance state. To reset the memristor, -1 V is applied as shown in Fig. 2, and the compliance current is increased to allow a higher current to pass through the device. From Figs. 1 and 2, it can be depicted that the fabricated devices exhibit electrochemical metallization (ECM) switching behavior, in which the creation of the conductive filaments is achieved by the ion migration resulted from the high electric field generated in the device. Moreover, during RESET operation, a synergic effect of joule heating takes place by allowing higher current to pass through the device and consequently achieving faster OFF switching for the memristor.

### Description of the memristor model and its fabrication process

**Figure 3:**
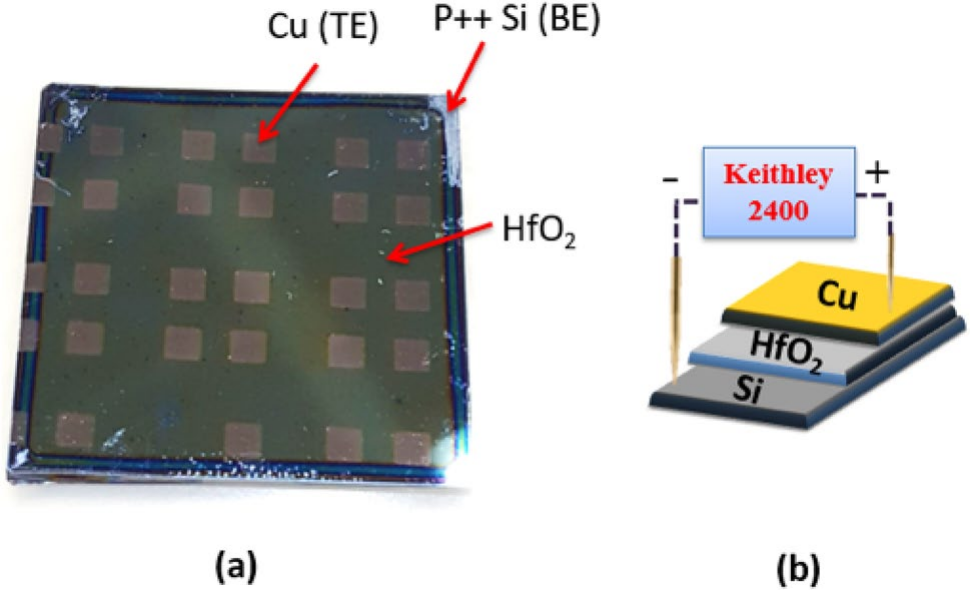
(**a**) Photo of one of the fabricated wafers with memristor devices (**b**) Schematic illustration of the HfO$_2$ memristor showing the stacked layers and the orthogonal alignment of the top and bottom electrodes.

**Figure 4:**
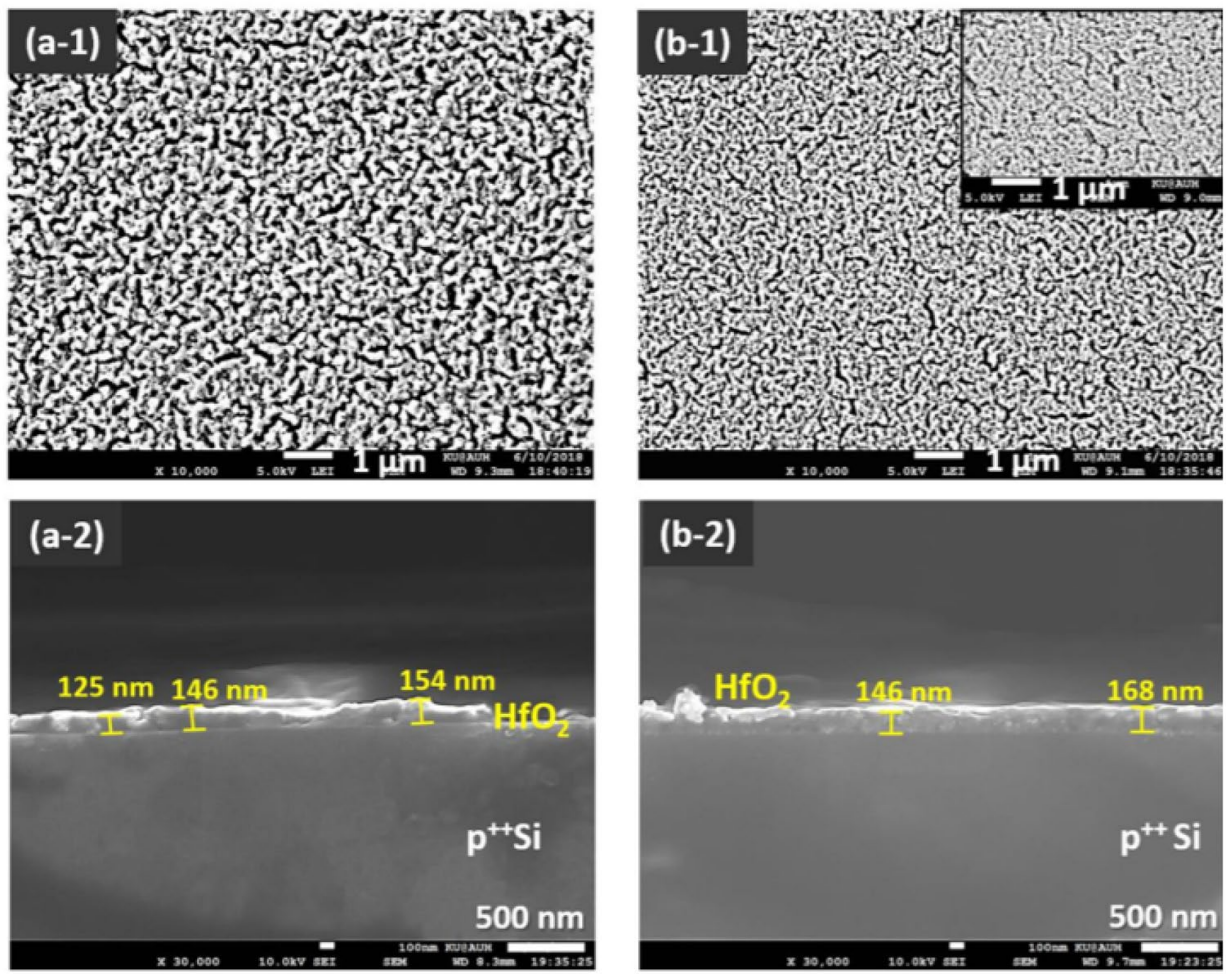
SEM images of the top views (**a-1**,**b-1**) and cross-section views (**a-2**,**b-2**) of HfO2/p++ Si regions from wafer samples A1 and A2, successively spin coated at a speed of 2000 RPM, using the same composite precursor mixture at room temperature. (**a-1**,**a-2**) sample A1; (**b-1**,**b-2**) sample A2. The inset in **b-1** displays another top view area of sample A2. The results show a denser surface texture of the oxide layer in sample A2 (**b-1**), implying a solution-aging factor. We can add the previous data to the paper if this is necessary.

The Cu/HfO$_{2-x}/p^{++}$Si device shown in Fig. 3 is fabricated in our lab using a low-cost sol–gel spin-coating method. Briefly, HfO$_{2-x}$ sol–gel solution is prepared by mixing hafnium isopropoxide isopropanol adduct (0.99 purity) with sulfuric acid, deionized water (*DI*), 2-methoxiethanol and polyvinylpyrrolidone (*PVP*). Contents are mixed between the addition of each new component and the HfO$_{2-x}$ precursor solution is left to stir overnight for *PVP* to dissolve. Ready HfO$_{2-x}$ solution is spin-coated on a heavily doped, $p^{++}$Si substrate pieces. Further, the sample is heat-treated in order to remove the organic residues from the oxide layer, originating mainly from the *PVP*. After heat treatment, a shadow mask sputtering step was performed to deposit Cu TEs using Q300T T sputtering tool by Quorum Technologies. Our memristor is cost-effective as it is based on thin film which uses spin coating for oxide deposition and only one metal deposition step. Usually, three deposition steps are needed to achieve a memristor stack; one for the bottom electrode, then the second is for the oxide layer and the third is for the top electrode. However, in this novel structure, the silicon wafer is utilized to act as a bottom electrode which eliminates one fabrication step and consequently results in a cost-effective device. Moreover, this device is compatible with mainstream CMOS technology and does not require any new materials nor masks. This lowers the cost of fabricating this device. The thickness of the deposited HfO$_2$ layer is in the range of nm ($\sim$ 150 nm) which leads to fast switching time in ns. This is considered great asset for high-speed Memristor-based PUFs. The scanning electron micros-copy (SEM) images that confirm the nm size of the used memristor device is shown in Fig. 4. The power consumption of the our proposed MR-PUF on average is 100 $\upmu$W. Note that the power consumption varies based on the used voltage set cycles in each iteration.

Keithley 4200-SCS Parameter Analyzer was used in the characterization of the I–V properties of the fabricated devices, no prior electro-forming was performed. The prepared memristors were electrically tested using a sweep cycling mode with a step of 0.05 V. $+\,3$ V and $-\,1$ V was applied onto the Cu electrode to set and reset the device, respectively.

The Memristor is highly nonlinear in voltage and time which makes it effective for security applications. The nonlinear mathematical model presented in Eq. (1) describes the behavior of memristive devices. The different parameters are defined as follows. $J_V$(x) is the current density at position x. $Q_v$ is the charge of oxide vacancy. $U_a$ is the activation energy of the ions. *f* is the escape attempt frequency. *a* is the hopping distance. *V* is the voltage applied across the memristor. $N_V(x)$ is the concentration of oxide vacancies at position x. $\alpha$ is a fitting parameter. $k_\beta$ is Boltzmann constant. *T* is the ambient temperature. *L* is the length of the memristor. *t* is the time duration of the applied voltage^42^.

1 $$\begin{aligned} J_v (x)= & {} 2q_vfa^2 \exp \left( \frac{-U_a}{K_\beta T}\right) \sinh \left( \frac{aq_v\alpha V/L}{2K_\beta T}\right) N_v(x) \nonumber \\&- q_v fa^2 \exp \left( \frac{-U_a}{K_\beta T}\right) \cosh \left( \frac{aq_v\alpha V/L}{2K_\beta T}\right) \frac{dN_v}{dx} \nonumber \\ \dfrac{dN_v}{dx}= & {} \frac{1}{q_v} \nabla .J_v \end{aligned}$$

### Proposed memristor unique switching behavior

**Figure 5:**
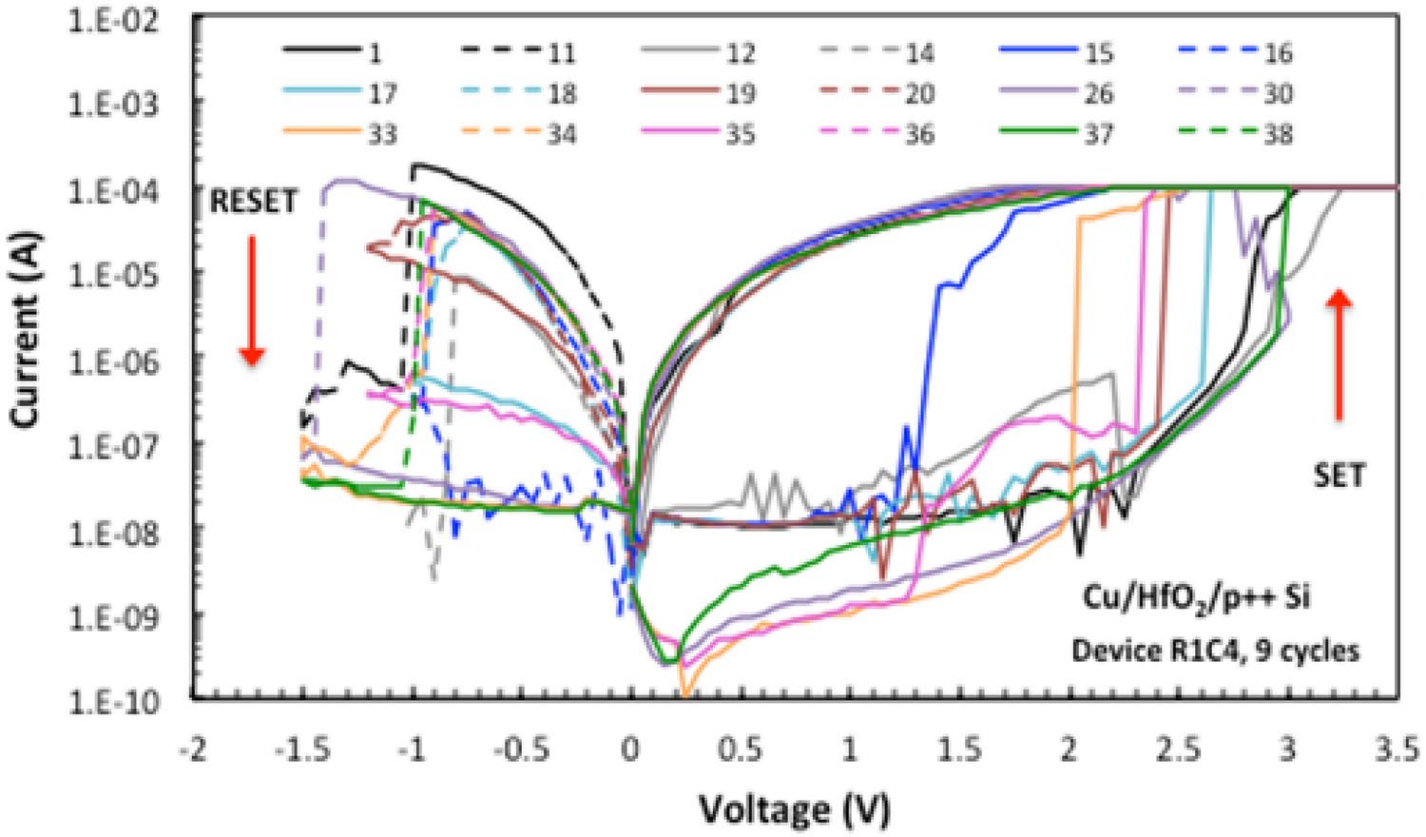
I–V characteristics obtained from one memristor device.

Depending on the material composition and the followed fabrication process, the filamentary-based switching mechanism can be highly probabilistic and uncontrolled which attributes to the final random sequence. Figure 5 presents consecutive I–V curves obtained by applying the same voltage sweep across the same memristor device. The data presented in Fig. 5 has been recorded in consecutive manners. However, some intermediate cycles are not shown for clarity and better readability of the figure.

The results shown in Fig. 5 are based on experimental data extracted from the wafer shown in Fig. 3. The stochastic behavior of the switching taking place in memristor devices is utilized in this contribution as the entropy source to generate the random output. The entropy source is inherited from the device ionic behavior that contributes to the device resistance switching^43,44^, in addition to the fabrication variations. Based on these factors, the memristor devices exhibit random variations in the fingerprint I–V characteristics from device to device, and from cycle to cycle within the same memristor cell. Although this is undesirable for memory, within computing and sensing applications, it is considered a desirable randomness asset for hardware-based security schemes^45–47^. Thus, in this work actual memristor devices are fabricated and the extracted switching parameters are used as the randomness source for the proposed security approach.

**Figure 6:**
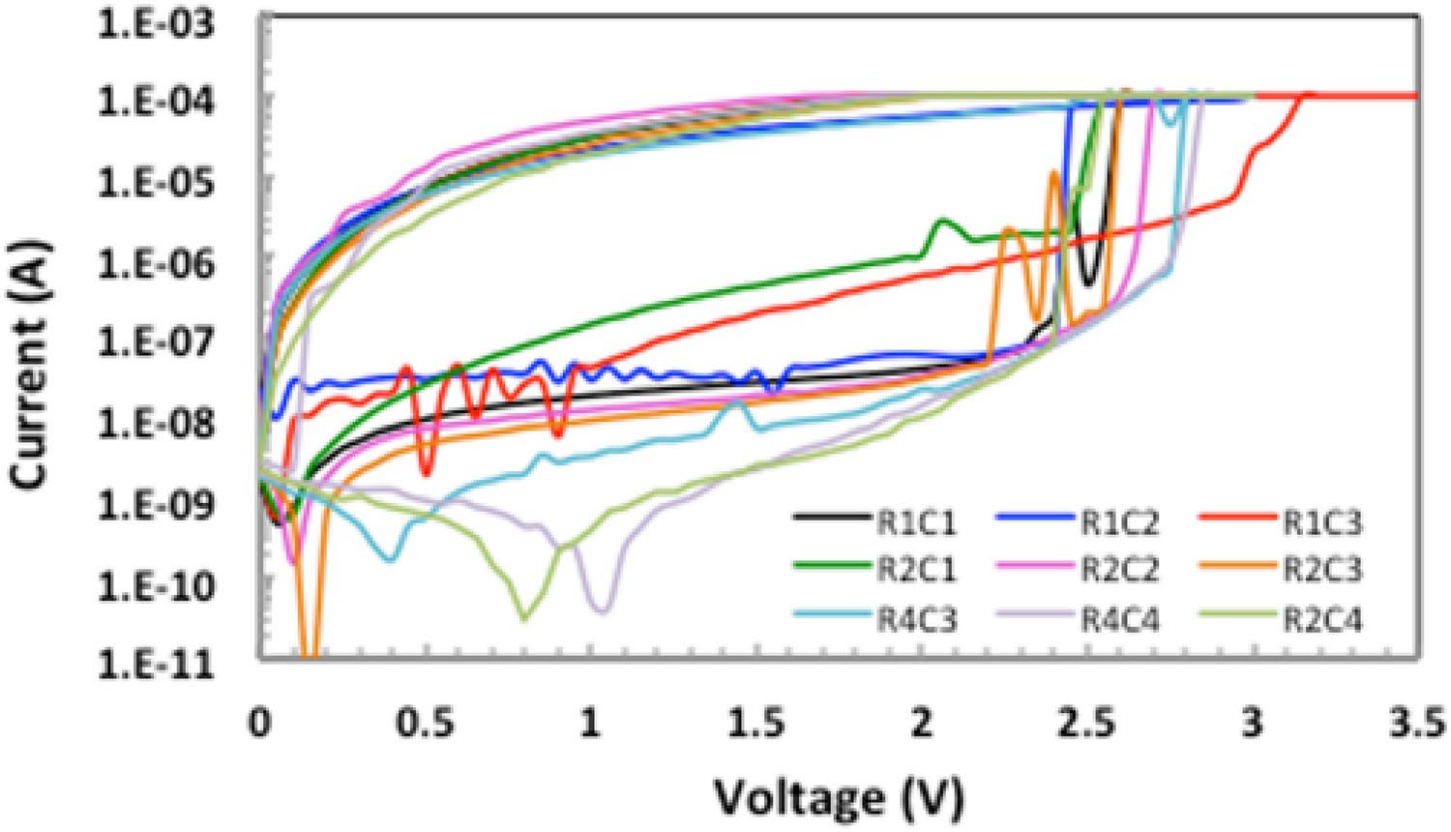
I–V characteristics obtained from identical memristor devices. Memristors fabricated on the same wafer using the same features and material compositions, each device has its own I-V values.

**Figure 7:**
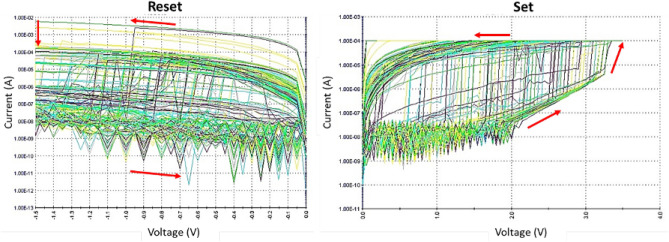
Endurance set-reset cycles of the fabricated MR-PUF.

Additionally, the uniqueness property can be verified by generating random sequences from identical memristor devices and calculating the inter-HD. As depicted in Fig. 6, although the memristors are fabricated on the same wafer using the same device features and material compositions, each device has its own unique output to the same challenge which leads to a distinguished true random bit sequence. More importantly, Fig. 7 presents the high endurance of our fabricated memristor which is related to the used HfO$_{2}$ material as a switching medium. This is consistent with many HfO$_{2}$-based memristors that are reported in literature^41^.

## Exploiting randomness from memristor switching behavior

This section investigates our testing algorithms for the fabricated MR-PUF. The random output generated is attributed to the set and reset operations of the fabricated MR-PUF to generate random responses. The natural variations in non-linear I-V curves, with the possibility of using voltage bias as a challenge (independent input bits), results in diversity restructuring sneak path currents providing random current values (Response). For every voltage in Figs. 5 and 6, there is a corresponding current and is different in every cycle ensuring the randomness of our responses. The initial random outputs are captured by our proposed MR by using Keithley 4200-SCS parameter analyzer by applying fluctuating voltages to the fabricated device. In parallel, using Keithley 4200-SCS Parameter Analyzer, we used a MATLAB model to record the output and test our MR-PUF signature using NIST statistical tests. Our MR-PUF passed the 15 NIST tests without any post-processing as shown in Table 4. In each test, the *P* value is given where the *P* value is the probability that a perfect random number generator would have produced a sequence less random than the sequence that was tested. *P* value $>\,0.01$ would mean that the sequence would be considered to be 99% random. On the other hand, a *P* Value $<\,0.01$ would mean that the sequence is 99% non-random.

### Randomness testing results

This property ensures the uniqueness of the PUF output. The PUF response must be unique, thus the probability for two devices having a similar PUF response is negligible. Each PUF response must be random and unpredictable.

For each input pulse to our TRNG MR-PUF , up to 32 random binary bits response can be collected. According to the NIST test protocol, 1M bits is collected and tested. Our TRNG MR-PUF’s response bits successfully passed the 15 NIST tests with a *P* value is greater than 0.01 and the pass rate exceeds the minimum value defined by NIST. The *P* value of the tests carried out are shown in Table 4. To further demonstrate the randomness of our MR-PUF, we compared the *P* values of the NIST Statistical results achieved by our MR-PUF with the NIST *P* value results of the Memristors presented in literature^31,34^ in Table 5.

**Table 4:** NIST 15 tests’ results.

NIST tests	*P* value	Bit string
Frequency Test	0.309	8740
Block Frequency Test	1	8740
Longest Run of Ones	0.55	8740
Runs	0.325	8740
Ranks	0.45	8740
Discrete Fourier Transform	0.731	8740
Serial	P1 = 0.18, P2 = 0.85	8740
Approximate Entropy	0.339	8740
Cumulative Sums	Pf = 0.263, Pr = 0.539	8740
Linear Complexity	0.8684	8740
Non-Overlapping Template	0.2918	1048576
Overlapping Template	0.1829	1048576
Random Excursions	0.1201	1048576
Random Excursions Variant	0.1153	1048576

**Table 5:** Comparison between the proposed Memristor NIST 15 tests’ results and the NIST results of the Memristors presented in literature^31,34^.

NIST tests	MR-PUF	D-Memristor^34^	RTN^31^
Frequency Test	0.309	0.447	is 0.987
Block Frequency Test	1	0.76	0.984
Longest Run of Ones	0.55	0.0424	0.987
Runs	0.325	0.042	0.993
Ranks	0.45	0.09	–
Discrete Fourier Transform	0.731	0.73	–
Serial	P1 = 0.18, P2 = 0.85	P1 = 0.74, p2 = 0.79	–
Approximate Entropy	0.339	–	–
Cumulative Sums	Pf=0.263, Pr = 0.539	–	–
Linear Complexity	0.8684	0.35	–
Non-Overlapping Template	0.2918	–	–
Overlapping Template	0.1829	0.59	–
Random Excursions	0.1201	–	–
Random Excursions Variant	0.1153	–	–

### Uniformity, diffuseness and uniqueness

We further assessed the randomness of our TRNG memristor PUF through evaluating vital standard metrics of randomness and reliability in cryptographic security primitives such as inter and intra-instance Hamming weight and Hamming distance. Uniformity is the measure of intra-response Hamming weight, and diffuseness is the measure of intra-PUF Hamming distance. These metrics evaluate the randomness of each PUF instance. Another important metric is uniqueness, which is the inter-PUF Hamming distance between responses to identical challenges to different PUFs. In order to evaluate uniformity and diffuseness, 100 different challenge sets are randomly applied to one MR-PUF. Each challenge consisting of $2^7$ bits, the $2^7$ single response bits are linked to form a 128 multiple bits response.

#### Uniformity

**Figure 8:**
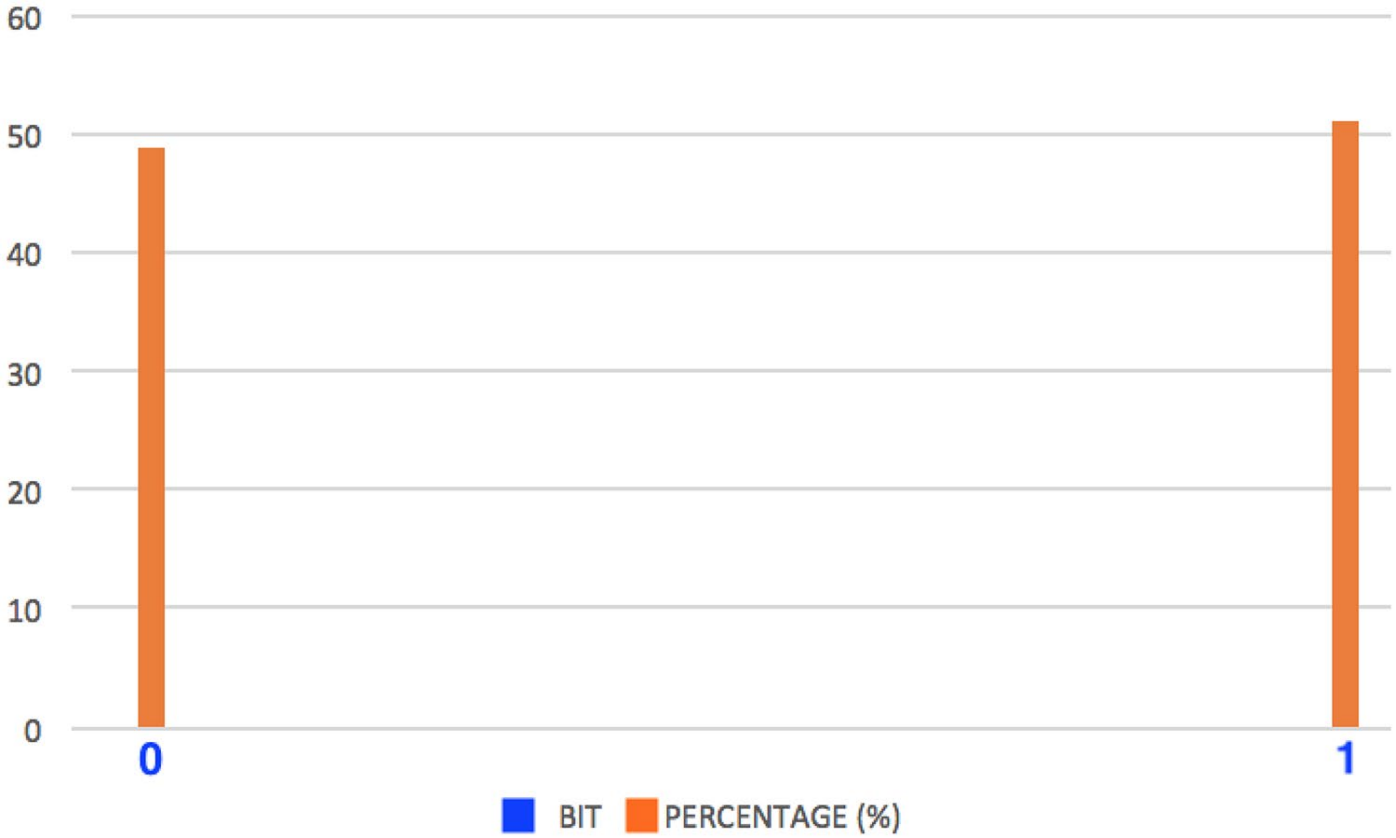
Probability of output logic ‘0’ and ‘1’ are near 50% (49% and 51% for logic ‘0’ and logic ‘1’ respectively).

Uniformity measures the percentage of ‘1’ and ‘0’ in responses of a PUF. Uniformity is achieved if the percentage is 50% for a truly random response. For our study, 100 different 128 bit challenges are send to one of our MR-PUFs and each 128 bits response vector acts as an identifier (ID) of a given MR-PUF. To evaluate the uniformity of our MR-PUF the percentage of ‘1’ and ‘0’ among all response vectors is calculated and illustrated in Fig. 8. From Fig. 8, it can be seen that both the probability of ‘0’ and ‘1’ are 48.9% and 51.1% respectively. Which is near to the ideal value of 50%. We carried out a comparison between our MR-PUF, R/W memristor^29^, Hybrid Memristor C-MOS PUF^30^ and rPUF^26^ in Table 6. It can be seen that our MR-PUF and R/W PUF have higher uniformity in comparison with Hybrid Memristor C-MOS PUF and rPUF .

**Table 6:** PUF performance uniformity metrics.

PUF types	Uniformity (%)
R/W Memristive PUF (8 bit)^29^	49.9
Hybrid memristor-CMOS PUF (128 bit)^30^	50.6
Re-Configurable PUF (rPUF) (128 bit)^26^	49.24
MR-PUF (128 bit)	49.9

#### Diffuseness

**Figure 9:**
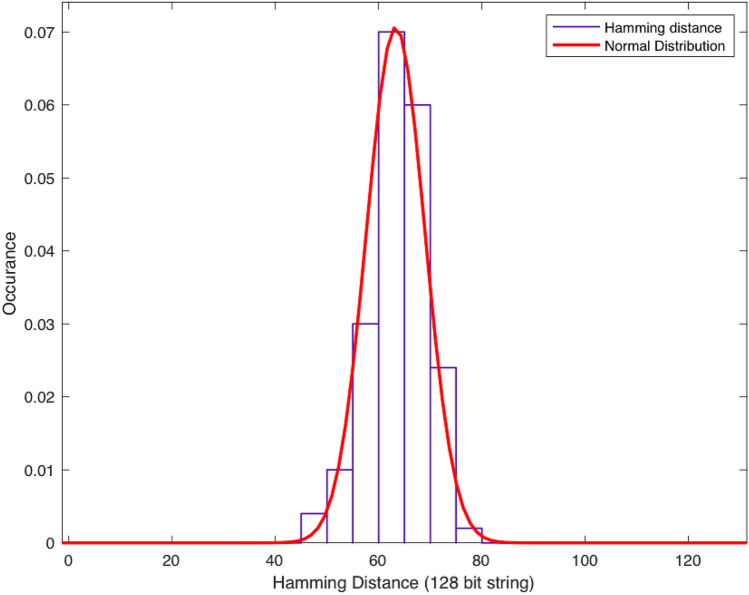
Hamming distance distribution for evaluating diffuseness: mean of HD is 63.43 which is 49.6% which is almost 50% the ideal value).

Normally, a PUF produces multiple bits responses. Diffuseness evaluates the difference between response vectors for different challenges applied to the same PUF. Diffuseness is evaluated by calculating the average of HD for all the possible response vectors generated by the same PUF. Diffuseness ideally is 50% in percentage which is half the response vector length. We calculated Hamming Distance (HD) between responses of our MR-PUF to determine the diffuseness of our proposed PUF. The diffuseness calculated for our study is 49.6% that is close to the ideal value of 50%, as shown in Fig. 9.

We carried out a comparison between our MR-PUF and rPUF^26^ in Table 7. The diffuseness of the rPUF is slightly higher than our MR-PUF; however, both are almost 50%.

**Table 7:** PUF performance diffuseness metrics.

PUF types	Diffuseness (%)
R/W Memristive PUF (8 bit)^29^	–
Hybrid memristor-CMOS PUF (128 bit)^30^	–
Re-Configurable PUF (rPUF) (128 bit)^26^	49.96
MR-PUF (128 bit)	49.6

#### Uniqueness

**Figure 10:**
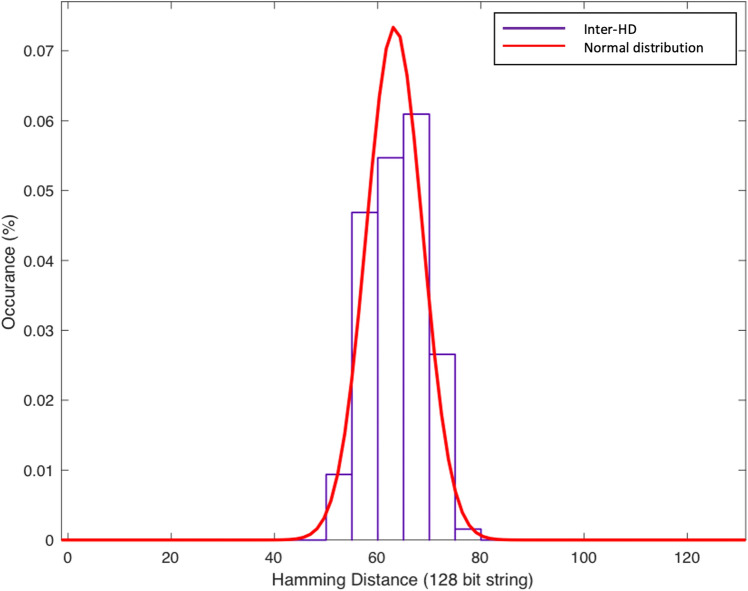
Hamming distance distribution for evaluating uniqueness: mean of HD is 63.2 which is 49.3% which is almost 50% the ideal value).

In the event of applying the same challenge to different PUFs, the response vectors from different PUFs should be different due to intrinsic variations of each PUF. This is a vital characteristic that evaluates the uniqueness of the information that can be extracted from a PUF. Uniqueness is measured by inter-HD. Ideally, the HD between the responses to the same challenge from different PUF instances should be 50%. In this paper, we used 100 different MR-PUF instances to evaluate uniqueness and the result is shown in Fig. 10 the mean of HD of MR-PUF is 63.3 bits out of the 128 bits response which is very close to the ideal value of 64 bits. We further compared our MR-PUF, R/W memristor^29^, Hybrid Memristor C-MOS PUF^30^, and rPUF^26^ in Table 8. It can be observed that the uniqueness of the four PUFs are very close in values and very close to the ideal value 50%.

**Table 8:** PUF performance uniqueness metrics.

PUF types	Uniqueness (%)
R/W Memristive PUF (8 bit)^29^	49.8
Hybrid memristor-CMOS PUF (128 bit)^30^	49.9
Re-Configurable PUF (rPUF) (128 bit)^26^	50.07
MR-PUF (128 bit)	49.3

### Repeatability

It is critical for a PUF to be used as an identification circuitry to always generate the same response when given the same challenge. While PUF uses physical units which are intrinsically chaotic, this criteria is difficult to meet precisely. In this study, we evaluated the repeatability of a response to a given challenge after hard resetting our MR-PUF. The HD count of the 128 bit response was equal to 0. Ensuring 100% repeatability of our TRNG MR-PUF.

Our MR-PUF has been verified in this section using NIST 15 tests. Additionally, the fundamental characteristics of our MR-PUF (uniformity, diffuseness, uniqueness and reliability metrics) have been evaluated. These tests verified that our model is hard to clone and resilient to adversary aims that predict responses to unseen challenges using a polynomial number of CRPs.

## TRNG based memristor applications

In this section, we will propose a design and analysis methods for TRNG based memristors as the one we have designed in cryptographic environments and high-level security environments as in advanced metering infrastructures (AMI). The merit of using the applications proposed (block cipher design, smart meter application) is to support the point around practicality and stability of TRNG design within cryptographic environment allowing security testing within an application environment regardless of the proposed level of complexity of the design.

### TRNG based memristor for cryptographic primitives

We are introducing a cryptographic design that will use our proposed MR-PUF. It is vital for ciphers that the communicating parties choose the key at random, without any possible bias or correlation between bits. The one-time pad’s main weakness to a nonrandom key might weaken the cipher to the point of making other attacks feasible^48^. If the cipher selects the key randomly, then a brute-force attack will take $2^N$ steps, where N is the key’s length. In the case of AES, this takes $2^{128}$ steps much more than the ability of even the fastest known computer. However, the assumption that the key is random plays an important role in avoiding a brute-force attack’s cost.

An adversary can learn a nonrandom key more quickly if it is known that the key’s bits are biased toward zero. Likewise, if the even positioned key bits tend to agree with the previous bit in the key, the search space is immediately cut by a square root down to $2^{N/2}$. Randomness might also impact the entire encryption process, not just key generation. An adversary could learn some information by simply observing the ciphertexts if encryption were deterministic. For example, if the sender transmits the same ciphertext twice, the adversary would observe that the same message was sent twice. In the case of a public-key scheme, a deterministic encryption technique provides the adversary with a way to detect if a given message is the encrypted one or not.

#### Block ciphers based on memristor based PUFs

**Figure 11:**
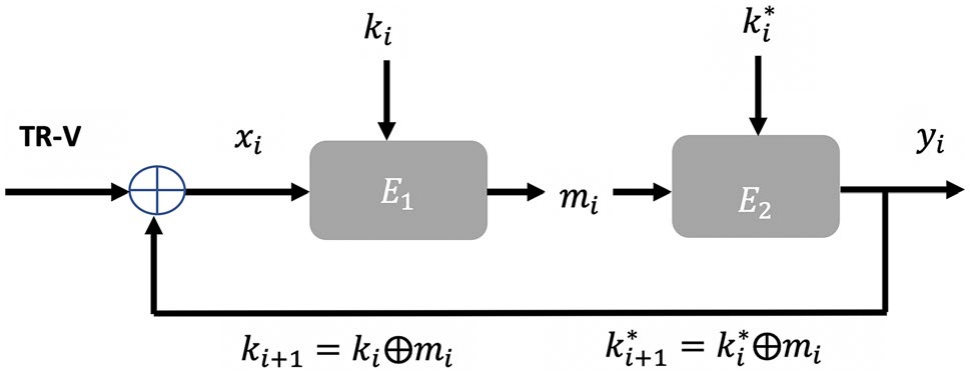
TRNG Cipher block.

We investigate our MR-PUF on block ciphers to achieve a true random encryption process. Our MR-PUF can be used in this section to convert block ciphers from PRNG to TRNG. Our TRNG Cipher block understudy is illustrated in Fig. 11. Figure 11 demonstrates a serial combination of two instances of a block cipher, denoted by $E_1$ and $E_2$, placed into the Cipher Block Chaining encryption mode. The input of the first block cipher is initialized to our MR-PUF response, a different MR-PUF response for each round, and each block cipher is initialized with its own master key, denoted *k* and $k^*$ respectively.

The execution of one round of our MR-PUF based block cipher is as follows: given the input of the first block cipher TR-V which is the random response of our MR-PUF, and the current value of the keys $k_i$ and $k^*_i$ used by the two block ciphers, an intermediate value $m_i$ is computed as $Ek_i(xi)$. The output of the TRNG is evaluated as $y_i = Ek^*_i(m_i)$. For the next round, the keys to be used by the block ciphers in the next round as $k_{i+1} = k_i$ $\oplus$ $m_i$ and $k^*{i+1} = k^*_i$ $\oplus$ $m_i$ and the new input for the first block cipher will be $x_{i+1} = TR-V$ $\oplus$ $y_i$, where the TR-V is a new response output of our MR-PUF. We refer to $k, k^*$ as the master keys and to $k_i$, $k^*_i$ as the running keys. The structure is generic thus that its input/ output/ key bit sizes are not specified (but identical): they depend on the actual block cipher chosen to instantiate the TRNG.

#### Security analysis

A comparison has been carried out to demonstrate and verify the randomness of the Cipher output when our true random MR-PUF response vector (TR-V) is used and when Pseudo-random vector (PR-V) is used. The output of the two block Cipher is tested by 8 NIST tests (due to the string bit size). The *P* value of each test is demonstrated to verify the randomness of the output Cipher string as shown in Table 9.

**Table 9:** NIST tests’ results for 256 bit string.

NIST tests (256 bit string)	TR-V *P* value	PR-V *P* value
Frequency Test	0.707660	0.211300
Block Frequency Test	0.766927	0.710185
Longest Run of Ones	0.000052	0.000018
Runs	0.017908	0.547623
Discrete Fourier Transform	0.818546	0.168669
Serial	P1=0.131016,P2=0.703588	P1=0.578957,P2=0.587870
Approximate Entropy	0.074202	0.438778
Cumulative Sums	Pf=0.687177, Pr=0.378538	Pf=0.378, Pr=0.267

### MR-PUF for advanced metering infrastructure (AMI)

In this section, a higher implementation of the MR-PUF was used on AMI systems. In an AMI system, most of the integrated circuit technologies including smart meters should support fundamental cryptographic competencies. Any smart meter should have its own secure cryptographic random value sequence. An AMI grid can consist of millions of smart meters. Accordingly, a large number of secure random keys in the range of millions are required. To avoid key disclosure in such high dynamic range and enhance the security of the entire communication system, utilization of a secure key management scheme is necessary.

**Figure 12:**
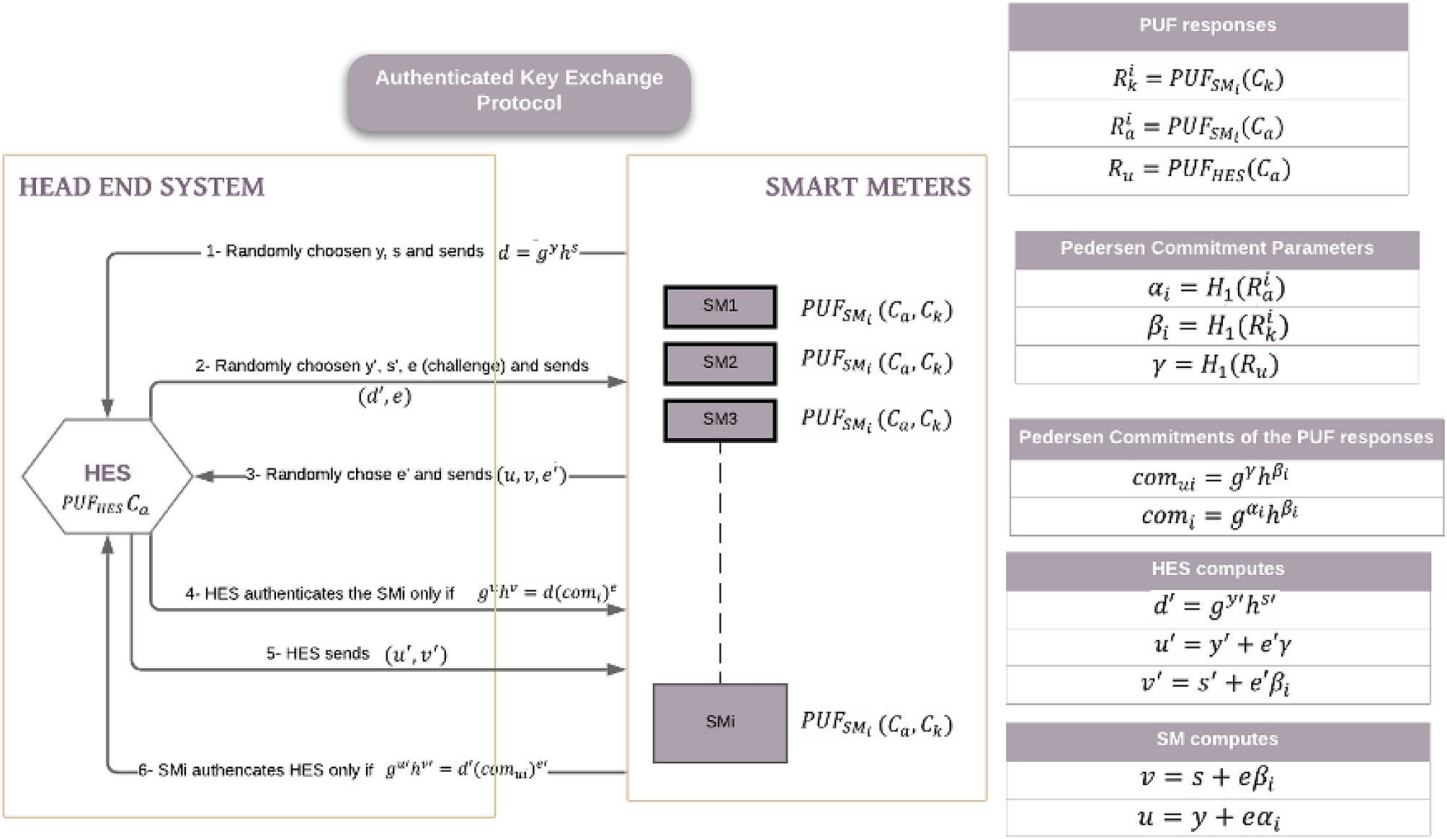
Authenticated Key Exchange Protocol Proposed.

In this paper, we explore an authenticated key exchange and message broadcasting protocols presented in^49^ exploiting our MR-PUF. In the explored scheme, the MR-PUF are embedded in both ends Head-End System (HES) and Smart Meters (SM) and used for generating the secrets random values. Okamoto Identification scheme a provably secure cryptographic protocol^50^, is employed in the authenticated key explored. This protocol meets the security requirements necessary for key management and mutual authentication. Utilizing this protocol, the SMs can authenticate and verify both the HES and the critical commands transmitted by HES. AMI Head-End System is positioned in the utility company, to gather data from SMs and send regulating commands, a two-way communication is required so the system can remotely manage configuration changes. Smart Meter is an electronic device that connects in two-way communication with the head-end system. It measures and records data such as energy usage and generation then transmits them to the HES in the utility.

Witness hiding identification protocols offer an adequate balance between security and efficiency. For example, for a protocol that accepts the prover successful only if it provides the complete private key. A cheating verifier may be able to extract some partial information on the private key, but the amount of information it can get is not sufficient for successful impersonation of the prover. Thus, the Okamoto protocol is a sufficient scheme as it satisfies the same properties of the honest-verifier. The important feature is that Okamoto’s protocol can be proved to be witness hiding. The prover’s private key is one such witness. An essential characteristic of Okamoto’s protocol is that it is witness indistinguishable, as the information seen by a random cheating verifier is independent of the particular witness used by the prover.

#### Cryptographic primitives used in the AMI

The utilized scheme under study consists of Initialization, Registration, and Mutual Authentication^49^. The Head-End System (HES) and the Smart Meter (SM) exchange a session key after authenticating each other.

- Initialization—In this phase, the Utility adjusts the system by executing the setup phase of the Pedersen Commitment scheme. Two challenges ($C_k$) and ($C_a$) are selected for which are applied to the PUFs implemented in the Head-End System (*HES*) and each Smart Meters ($SM_i$).
- Registration—In this part shown in algorithm 1, some information must be shared between the *HES* and $SM_i$ before executing the protocol. Thus, the following procedures are executed in the registration phase. As shown in Fig. 12, the first two challenges $C_k$ and $C_a$ are given to $PUF_{SM_i}$ generating the corresponding responses $R^i_k$ and $R^i_a$. The same procedure is done with the $PUF_{HES}$ given $C_a$ as a challenge and producing $R_u$. For computing the commitments, the cryptographic hash function $H_1$ is applied to all of the PUF responses. Thus, the parameters $\alpha _i$, $\beta _i$, and $\gamma$ are produced. Where $\alpha _i = H_1(R^a_i)$, $\beta _i=H_1(R^i_u)$, and $\gamma = H_1 (R_u)$. Then $\beta _i$ and $h^\gamma$ are stored in *HES* and $SM_i$ respectively. These parameters are used to compute the Pedersen commitments of the PUF responses as follows $com_i=g^{\alpha _i}h^{\beta _i}$, $com_{ui}= g^u h^{\beta _i}$. $com_i$ and $com_ui$ are stored in *HES* and $SM_i$, respectively.
- Mutual Authentication- In this step, Okamoto protocol is used for mutual authentication between the *HES* and $SM_i$. As shown in Fig. 12 and algorithm 2, they elaborate what is done in this phase. First, $SM_i$ chooses y, s randomly and sends $d=g^yh^s$ to the *HES*. Then, *HES* randomly chooses $y', s'$ and e (as a challenge), and computes $d'=g^{y'}h^{s'}$. Second, *HES* returns a tuple ($d',e$) to $SM_i$. Third, $SM_i$ chooses a random value $e'$, computes $v=s+e\beta _i$ and sends the tuple (u,v,e’) to the *HES*. Fourth, *HES* verifies $SM_i$ only if $g^uh^v=d(com_i)^e$. Fifth, *HES* computes $u'=y'+e'y, v'=s'+e'\beta _i$ and sends the tuple (u’,v’) to $SM_i$. The last step, $SM_i$ verifies *HES* only if $g^u=d'(com_{ui})^{e'}$.

The initialization, registration and mutual authentication algorithms have been implemented on MATLAB(*Mathworks*) using Okamoto Protocol and our fabricated MR-PUF output. The head-end system was able to verify all the Smart Meters implemented on MATLAB, and the Smart Meters were able to verify the Head-end system. 
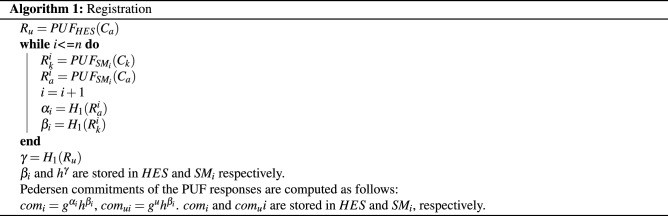

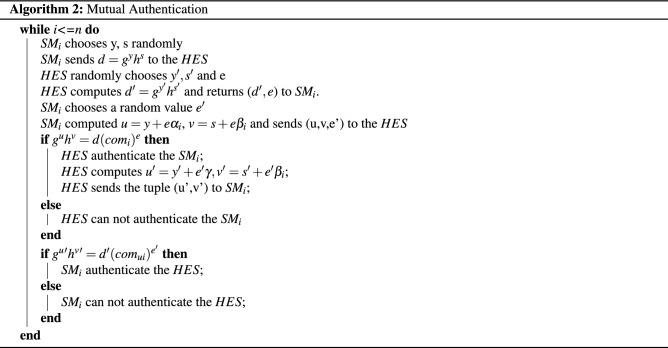


#### Security analysis of our model

To ensure the randomness of the $PUF_{HES}$ and $PUF_{SMi}$ responses, NIST 800-22 statistical tests have been used to verify the randomness of $PUF_{HES}$ and $PUF_{SM1}$, verifying the randomness of our proposed MR-PUF as shown in Tables 10 and 11. Exploiting our MR-PUF, hash function, and Okamoto protool we used MATLAB to analyze the security of communication in the advanced metering infrastructure understudy. We enhanced the uniqueness of the MR-PUF response to a specific challenge, through applying a hash function to PUF responses to avoid colliding users from obtaining keys which they are not allowed to obtain individually ensured the high security for the key. Moreover, there is no need to store all the key materials as the session key is generated when needed and no other entity has used it.

**Table 10:** NIST 8 tests’ results for $PUF_{HES}$.

NIST tests $PUF_{HES}$ output bit string	Ru *P* value
Frequency Test	0.347413
Block Frequency Test	0.853513
Longest Run of Ones	0.012301
Runs	0.549466
Discrete Fourier Transform	0.339761
Serial	P1 = 0.451674, P2 = 0.268390
Approximate Entropy	0.504039
Cumulative Sums	Pf = 0.685193, Pr = 0.404218

**Table 11:** NIST 8 tests’ results for $PUF_{SM1}$.

NIST tests $PUF_{SM1}$ output bit string	Ra *P* value
Frequency Test	0.382625
Block Frequency Test	0.97163
Longest Run of Ones	0.306104
Runs	0.630535
Discrete Fourier Transform	0.298631
Serial	P1 = 0.633505, P2 = 0.454436
Approximate Entropy	0.711780
Cumulative Sums	Pf = 0.320593, Pr = 0.475339

## Conclusion and future work

In our study, we presented the current research work on a low-cost, high endurance, and high speed Cu/HfO$2/p^{++}$Si MR-PUF relying on nano-particle dynamic simulation and analytical assessments. The thickness of the deposited HfO$_2$ layer is in the range of nm ($\sim$ 150 nm), thus, allowing fast switching time in ns. The HfO$_2$ material is used as a switching medium of our MR-PUF providing high endurance due to the high stability of the material. Leveraging the memristor’s two level variations, we achieved a unique, reliable, and irreversible MR-PUF output. We tested different Cu/HfO$2/p^{++}$Si MR-PUFs, the same challenge gave different responses ensuring the uniqueness of our MR-PUF. The repeatability of each MR-PUF has been tested and our proposed MR-PUF has proven consistency as each MR-PUF reproduces the same response to the same repeated challenge. Furthermore, MR-PUF passed 15 NIST 800-22 statistical tests without any post-processing techniques . We investigated exploiting MR-PUF random output vector to achieve a TRNG block ciphers model and verified the randomness of the MR-PUF based Ciphers’ output using NIST tests.

We explored the comparison between the MR-PUF based Block Ciphers and the original Block Cipher design to demonstrate and test the randomness of our design. Moreover, in this paper we employed the MR-PUF in an authenticated key exchange and mutual authentication between the HES and SMs in an AMI. The AMI based on our MR-PUF met the essential security requirements as it passed the NIST tests and the mutual authentication was verified for both ends (HES and SM). To ensure that the session is always unique, hash functions were applied to PUF responses to avoid colluding users from obtaining keys which they are not allowed to obtain individually. We explored this by storing in each of the smart meters only the hashed values $h^\gamma$ and computing the other hashed parameter $\beta _i$ at the SM side by applying the hash function of the response of $PUF_{SMi}$ which is unique for each SM. Our testing included simulating the environment and the verification testing of authentication to each side SM and HES.

As a future work, this research is meant to explore different attack scenarios of the proposed PUF architecture and it applications in different environment. This includes attacks built to influence the randomness of the proposed PUF circuits by introducing different voltage values to specific devices in search of collisions or reduce the write time in the memristor-based PUF to influence the repeatability feature. This in essence renders the PUF output no longer unclonable. Furthermore, improvements to the blockcipher model of application will be analyzed to explore practical and lightweight variations of the model. Additionally, AMI like any other smart grid application is exposed to several threats. The two major attacks targeting AMI systems are (a) attackers aiming to gain access to confidential data from users so they can infer the scheduled unit’s behaviour to target them for physical attacks; and (b) users may attack and alter the energy usage data to induce energy theft. Additional, exposure of these attacks is to be considered within our future work.
